# Medical and Philosophical Intelligence

**Published:** 1812-12

**Authors:** 


(To be continued.)

### MEDICAL AND PHILOSOPHICAL INTELLIGENCE.

PHILOSOPHICAL SOCIETY OF LONDON.—During the last month, the attention of the society has been directed to two lectures on the subject of Light and Heat, by Mr. William Henley.

As the ancient theories of light and caloric had undergone so much discussion, and as other new ones had lately been adopted and supported by several illustrious philosophers, it became an interesting subject for the investigation of the society.

In the lecture on light, Mr. H. gave a very able analysis of the curious experiments of Mr. Malus, which confirmed his opinion of the polarity of light; and, after the most impartial review, he felt perfectly satisfied with the opinions of Sir Humphrey Davy on this point.

On the subject of caloric, he entered at much length, and gave an abstract of the different theories, from that of Zeno to those of the present time. He dwelt more particularly on the two theories maintained by the philosophers of the present day, and his arguments were directed principally against the *material* theory; that which supposes caloric to be a peculiar and subtle fluid, capable of perverting, in a greater or less degree, every kind of matter, and of counteracting, by its repulsive power, the attraction of cohesion, an opinion adopted almost universally by the chemists. Mr. H. brought forward a number of facts which it could not satisfactorily explain, but which favored, in the most happy manner, that hypothesis which accounts for the phenomena of heated bodies by supposing the particles of matter to be endowed with a power of repulsion, and that the effects of heat are only attributable to a rapid motion of the minuter particles of matter, rather of a vibratory kind, or of one round their own axis, an opinion now warmly supported by Sir H. Davy. The theory was supported by the lecturer by a number of well chosen experiments, which, though not able, perhaps, of carrying conviction to the supporters of the material theory, are yet deserving of their attention, as affording a proof of the impossibility of satisfactorily accounting for all the phenomena of heat by any one single theory.

---

At the adjourned *General Meeting of Apothecaries*, held on the 20th instant, at the Crown and Anchor, Strand, which was numerously and respectably attended, (Mr. BURROWS in the Chair,) it was

Resolved nem. con.—1st. That, although the excessive price of glass led to the original appointment of the Committee of Apothecaries, yet, upon mature deliberation, it is determined that the consideration of this subject be for the present suspended.

Resolved nem. con.—2dly. That the improved education of the regular apothecary has much advanced the importance and utility of his character, and been highly advantageous to the public; but that his services have been by no means sufficiently remunerated, nor his respectability advanced in proportion.

Resolved nem. con.—3dly. That a large body of pretenders have assumed the character and functions of the regularly-educated apothecary, to the great detriment of the public health.

Resolved nem. con.—4thly. That these evils have arisen from the want of a proper superintending body.

Resolved nem. con.—5thly. That the executive of the Royal Colleges of Physicians and Surgeons, and the Society of Apothecaries, be requested to concur and unite in an application to parliament for an Act for the improvement and better regulation of the Practice of the Apothecary throughout England and Wales.

Resolved nem. con.—6thly. That the present committee be invested with full powers to obtain the objects of this meeting by such means as, in their judgment, may be thought proper.

Resolved nem. con.—7thly. That the committee report their proceedings from time to time; and that a general meeting be again called, should a requisition for that purpose be signed, and sent to the present chairman, by twelve subscribers.

Resolved nem. con.—8thly. That Mr. Parkinson, Hoxton-square, be appointed one of the committee.

Resolved nem. con.—9thly. That country practitioners be requested to form distinct committees, to co-operate and correspond with the London committee on the means best adapted to promote the general and local interests of the profession.

Resolved,—10thly. That, to defray the expenses of an application to parliament, subscriptions of two guineas be solicited from each apothecary in England and Wales, to be paid into the banking-house of Messrs. Gosling and Co. No. 19, Fleet-street, or to any one of the Treasurers or members of the Committee.

Resolved nem. con.—11thly. That Messrs. Burrowes, Simons, and Field, be appointed Treasurers.

Resolved nem. con.—12thly. That all communications to the committee be directed (postage free) to the Secretary, No. 13, Great Russel-street, Bloomsbury.

Resolved nem con.—13thly. That the thanks of the meeting be given to the committee for their very able Report, and for their unremitting attention to the objects for which they were appointed.

*George Man Burrows*, Chairman.

Resolved nem. con.—14thly. That thanks be given to the chairman for his impartiality, zeal, and attention, in discharging the duties of the chair. *John Powell*, Secretary.

*Note*—The committee, aware that it will be gratifying to those who take an active interest in promoting the objects of the above Resolutions, to find them promptly and extensively supported by the profession, intend publishing regularly the names and residences of the subscribers.

---

*Spontaneous Combustion in Manufactories, &c.*—The frequent accidents in our manufactories by fire, have excited the attention of scientific men. We find two papers published on this subject; one in the New York Medical Repository, by Dr. Seybert, member of Congress, a distinguished chemist and mineralogist; the other in the Philadelphia Emporium, by Dr. Coxe, Professor of Chemistry. It appears, from their researches, that a multitude of subtances are capable of spontaneous inflammation, and that others evolve gaseous fluids which suddenly inflame on the approach of fire. A curious instance of the latter is related from Count Morozzo. While a baker's boy in Turin was turning over some flour in a small room where a lamp was suspended, a sudden inflammation took place, followed by so violent an explosion, that the windows and window-frames in a shop below were thrown into the street. Repeated instances of a similar nature have occurred.

Among the list of articles mentioned by Dr. Seybert as having taken fire without the approach of flame, are the following:

Candle-wick made of hemp-yarn, accidentally impregnated with oil.

Cotton goods on which linseed-oil had been spilt.

Roasted bran in a linen cloth.

Wet hay, corn, and madder; especially if any portion of iron should be intermixed.

Sail-

Sail-cloth, smeared with oil and ochre, took fire in a magazine at Brest.

New cloth; and fire-wood soot immersed in hemp-oil varnish.

Two Russian frigates were destroyed by the spontaneous inflammation of German lamp-black.

Vegetables boiled in oil or fat, and left to themselves, after being pressed.

Heaps of linen rags in paper manufactories.

Pyrites, and cinders from the furnaces of glass-works, when exposed to a moist atmosphere.

Cuttings of iron, which had been previously immersed in water.

The Pantheon in London was destroyed by the inflammation of a paint made of Derbyshire woad, used in painting the scenery.

It is probable that many other substances will be found capable of undergoing the same process. We are told of the spontaneous combustion of brandy-drinking men and women, especially of the latter. Prudent people will be inclined to wait for better evidence than we have yet seen, before they give credit to these last accounts, even when they come from learned societies. The others are sufficiently authenticated, and ought to inspire those concerned in manufactories with circumspection.—*New-England Journal of Medicine and Surgery.*

---

*Interior of Africa.*—A letter from the German traveller, Routgen, who has announced an intention of penetrating into the centre of Africa, and reaching, if possible, the celebrated city of Tombuctoo, has been received by his brother, and published at Neuwied, in Germany. It is dated the 21st July, 1811. In it he states, that he had fortunately found a companion for his travels in the person of a German, who had turned Mussulman at Tetuan, who has traversed the Barbary States in all directions, and had made the pilgrimage to Mecca. He had lived at Jamba, in Africa, as a coffee-house keeper, and at Jauni, as a physician. At Constantinople he had superintended the gardens of a Pacha. He acted as gardener to a merchant at Mogadore, where Routgen found him, and took him into his service, but treated him rather as a friend than a servant. Routgen saw at Morocco preparations making for the setting out of a caravan, which was to reach Tombuctoo by Tafilet and Tunt, and immediately formed a resolution to join it. He caused it to be reported at Mogadore, that, disgusted with the bad treatment he had received at Morocco, he meant to repair to Tangiers, and from thence embark for Gibraltar. This furnished him with a pretext for purchasing a mule and every other necessary, and he secretly procured some Moorish garments; for he observes, it is impossible to convey an idea of the violent hatred of the Moors against Christians, which arises in a great degree from the difference of dress. When his preparations were finished, he went with some Christians on a party of pleasure, to a mountain about six miles off, and they, at their return, would give out that he was on his way to Tangiers; but, as soon as he was alone with his companion, he meant to clothe himself in his Moorish garb, and enter the great road which

 leads leads from Tafilet to Morocco, whence he would reach Deminit, and join a caravan which passes there about that time. With it he would cross Mount Atlas, and the burning plains of Tafilet. At Tafilet he meant to reside with a German renegado, for there are many Germans there. He also expected to meet with a German at Tombuctoo, where he proposed to remain six months. It was his intention to penetrate towards the south, and, if possible, to reach Wesemb, or the Cape.

---

A short time since, immediately after an unusually high tide, a fossil crocodile, 17 feet in length, was discovered under the cliffs between Lyme Regis and Charmouth. It was dug out of the cliff nearly on a level with the sea, and 100 feet below the surface of the earth.

---

A woman of the commune of Sonnegham, 26 years of age, after being fifteen months pregnant, had a full-grown fœtus extracted from the left ovarium, by M. Kluyskons, surgeon, in September last. The fœtus was found in a state approaching to putrefaction, and the woman perfectly recovered.—*Journal de Paris, Nov. 9*, 1812. It should seem from this short notice, that an abscess pointed in the left side, which being cut into, the fœtus was discovered and extracted. M. Kluyskons is expected to publish the details of this unusual case.

---

*Deaths by Small-pox weekly, from the London bills.*

| | | | |
|---|---|---|---|
| Oct. 13, . . . . | 55 | Oct. 27, . . . . | 50 |
| 20, . . . . | 56 | Nov. 3, . . . . | 57 |

---

A new Philosophical Journal, by Dr. Thomas Thomson, author of "A System of Chemistry," &c. will be commenced with the ensuing year, under the title of "Annals of Mechanical Philosophy, Chemistry, Agriculture, and the Arts."

---

Mr. Callow, so well known to the medical profession, for his extensive and unequalled Medical Library, has in the press a Catalogue of his valuable collection. Among many curious, rare, and elegant, works, contained in this catalogue, we have noticed the following: Alsaharavii, *Liber Theoricæ, nec non Practicæ, jam summa Diligentiâ, et curâ depromtus in Lucem per S. Grimmium. Aug. Vind.* 1519.—Camper, *Icones Herniarum a Soemmering; Demonstrationum Anatomica, &c. &c.*—Celsus, *de Medicina, Editio Princeps*, 1478.—Hippocrates et Galeni, *Opera omnia, Gr. et Lat. per Charterium.*—Mascagni, *Vassorum Lymphaticorum Corporis Humanii, Historia et Ichnographia.*—Soemmering, *Abbildungendes Menschilichen Auges.*—Scarpa, *Anatomicæ Disquisitiones de Auditu et Olfactu;* and, *Tabulæ Neurologicæ ad Illustrandum Historiam Anatomicam.*—Clowes' scarce work, the "Briefe and Necessarie Treatise, touching the Cure of the Disease called Morbus Gallicus," &c. 1585.—*Encyclopedie Methodique Chirurgie, par Mess. Roche et Radel.*—Valentine Greatrack's Brief Account, &c. by Stubbe, 1666. —Haller's *Bibliotheeas*, and Ploucquet's *Literatura Medica*, &c.

Mr.

Mr. Stevenson intends very speedily to publish, in one volume 8vo. "Practical Remarks on Cataract;" which will contain, besides considerable additions to the contents of his ingenious communications on that interesting subject, inserted in the two last Numbers of our Journal, a History of the Symptoms, together with a description, and a highly-finished engraving, of his improved Speculum Oculi.

---

Mr. Hebb, of Worcester, has in the press a Translation of Corvisart's work on the Diseases and Organic Lesions of the Heart and great Vessels; in which the author, after taking a philosophical and physiological view of his subject in a preliminary discourse, divides the diseases of the heart and large vessels into five great classes.

In the first class, he treats of the Diseases of the membranous Coverings of the Heart. Under this head are included pericarditis, adhesion of the pericardium to the heart, and hydrops pericardii.

In the second class, of Diseases of the muscular Substance of the Heart. Aneurism active and passive. Induration and ossification of the heart, and its conversion into a fatty substance.

In the third class, of Diseases of the tendinous and fibrous Parts of the Heart. Induration of the right and left auriculo ventricular orifices and ossification of the valves. Excrescences or vegetations on the valves.

In the fourth class, of Diseases of various Tissues of the Heart. Carditis, rupture of the heart, tumors of the heart, and other unnatural conditions of that organ. Perforation of the septum of the ventricles or auricles. Non-closure of the foramen ovale after birth. Closure of the foramen ovale in the fœtus.

In the fifth class, of Aneurisms of the Aorta, true and false. The whole illustrated and enriched by numerous cases and dissections.

This Translation is made from an enlarged and improved edition, published by Corvisart, in 1811, and lately imported into this country.

---

The first part of Dr. Barton's *Flora Virginica*, has been published in America. This is a work to which the naturalists of Europe will look with great expectations, and which the exertions and accuracy of Dr. Barton will, probably, not disappoint.

---

Dr. Adams will commence his Spring course of Lectures on the Institutes and Practice of Medicine early in January 1813, at his house, No. 17, Hatton Garden.

---

*Lectures on the Anatomy, Physiology, and Pathology, of the Eye and Ear*, by Mr. Stevenson, surgeon, &c.—This course will commence with a description and demonstration of every part of the complex structure of the Eye and Ear, which will be illustrated by appropriate anatomical preparations and drawings, as well as by occasional references to such subjects of comparative anatomy as, by displaying a similar but more distinct organization, tend to elucidate the mechanism of our own.

In treating of their Physiology or healthy actions, particular inquir quir quiry will be made into the relative importance of each individual structure to the economy of the whole apparatus. This will lead to assign the reasons, why some parts may be impaired, or even destroyed, without materially injuring the functions of the organs;—to account for their greater or less degree of perfection in the several classes of created beings;—and to explain the causes of their adaptation to the different media in which they are respectively destined to be exerted.

Lastly, the Pathology of the Eye and Ear will comprehend an investigation into their morbid structure and deranged functions. To this most important practical part of the Lectures, an adequate portion of time will be devoted, to admit of a clear developement of the nature, causes, and symptoms, and a full explanation of the most approved modes of treating their diversified, and frequently complicated, ailments. The requisite surgical operations, together with the best method of porforming them, will likewise be explicitly detailed. Cataract, confessedly one of the principal among the interesting series of ocular diseases, will also obtain a share of consideration proportioned to its acknowledged importance; the discussion of which will include a description and an analysis of the respective merits and defects of the several processes that have been adopted for its cure, from the earliest down to the present period.

The introductory Lecture, to which all professional gentlemen and students may have free admission, will be delivered January the 26th, at eight o'clock in the morning.

---

Dr. Clough, Berner's-street, will commence his ensuing course of Lectures on the Science and Practice of Midwifery, including the Diseases of Women and Infants as connected therewith, on Monday the 4th of January, at half past ten in the morning, and at seven in the evening. Students, when properly qualified, will be amply provided with cases to attend in rotation, which are governed by the number admitted at the institution; and their views greatly forwarded by weekly examinations of the preceding lectures. A prospectus may be had as above, and of all the medical booksellers.

---

Mr. Singer will commence his Lectures at the Scientific Institution, Princes'-street, Cavendish-square, towards the close of December. The highly interesting subject of Electricity, entirely omitted in the arrangement of last year, will, we are informed, constitute the leading subject for the ensuing season. To the powerful apparatus before employed by Mr. Singer, many important additions have been made: amongst others, we have noticed a series of De Luc's electric columns, of unparalleled extent, and of various novel and effective combinations; and also an entirely new voltaic apparatus of a thousand double plates.

METEO-

