# Corrigendum

**Published:** 1813-01

**Authors:** 


### *CORRIGENDUM.*

In the notice of Mr. Stevenson's Lectures in our last Number, for "*eight o'clock in the evening,*" read *eight o'clock in the morning.*

